# A74 SURVEILLANCE IMAGING FOLLOWING COMPLETELY RESECTED GASTROENTEROPANCREATIC NEUROENDOCRINE TUMORS: A SINGLE CENTER AUDIT OF LOCAL PRACTICE PATTERNS

**DOI:** 10.1093/jcag/gwab049.073

**Published:** 2022-02-21

**Authors:** J Iannuzzi, C Yeo, S Sawhnry, J Pasieka, V Parkins, D Ruether, D Chan, Z Albalawi, K Lithgow

**Affiliations:** Medicine, University of Calgary, Calgary, AB, Canada

## Abstract

**Background:**

Neuroendocrine tumours (NET) are a heterogenous group of neoplasms that secrete peptides and neuroamines. For patients with potentially malignant gastroenteropancreatic (GEP) NET, surgical resection represents the only curative option. GEP NETs are characterized by long periods of disease-free survival and time-to-recurrence following surgical resection. Clinical guidelines recommend surveillance with cross sectional imaging, either CT or MRI, for at least 10 years.

**Aims:**

The purpose of this study was to characterize local practice patterns of imaging surveillance (modality, frequency, and duration of follow-up) and how this compares to guideline recommendations.

**Methods:**

We retrospectively reviewed clinical and imaging records from patients diagnosed with well-differentiated GEP NET at our center from January 2005 to July 2020 inclusive. Eligible cases were identified by a data analyst from the Alberta Cancer Board with each case being manually screened for eligibility. Exclusion criteria included patients with metastatic disease at presentation, G1 appendiceal NET < 1 cm, R0 G1 T1 rectal NET, and insulinoma. Location of primary NET, modality of surveillance imaging, date of test and duration of follow-up collected. The mean number of surveillance scans per person and per person-year follow-up based on the location of the primary NET were calculated.

**Results:**

A total of 387 cases were initially retrieved with 62 eligible cases identified. The mean length of follow-up was 71 months (range 8 to 147). The mean number of surveillance scans was 7 (range 2 to 14) and the mean number of surveillance scans per person year was 1.1. Frequency of surveillance scans per year of follow-up did not differ based on the location of the primary tumor (p=0.966). Imaging modalities included cross sectional imaging (MRI and contrast enhanced CT) and nuclear medicine imaging (octreotide, MIBG, F-18 FDG-PET, and Gallium-68 DOTATATE PET CT). Most commonly, cross-sectional imaging was performed with CT or MRI representing 38% (n=166) and 39% (n=170) of all surveillance respectively. Nuclear medicine imaging was used in 15% (n=61) of surveillance scans and 3% used combined cross-sectional and nuclear medicine. Amongst cases with resection date *>*10 years (n=8) mean length of follow-up was 119 months (9.9 years).

**Conclusions:**

Frequency and modality of imaging at our center is generally in accordance with current clinical guidelines, though the role of nuclear medicine imaging in this setting has not been established. CT and MRI were utilized equally during surveillance. The burden of these modalities in terms of radiation exposure and cost warrants further evaluation.

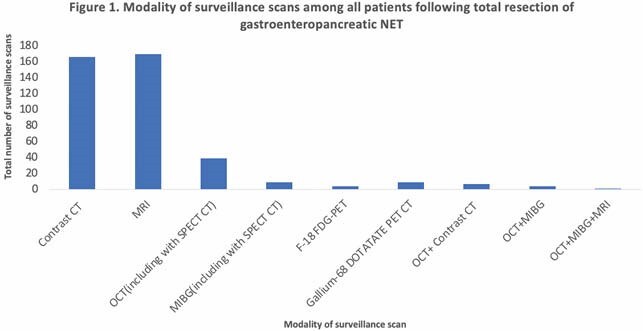

**Funding Agencies:**

None

